# Diamondoid Characterization in Condensate by Comprehensive Two-Dimensional Gas Chromatography with Time-of-Flight Mass Spectrometry: The Junggar Basin of Northwest China

**DOI:** 10.3390/ijms130911399

**Published:** 2012-09-12

**Authors:** Shuifu Li, Shouzhi Hu, Jian Cao, Ming Wu, Dongmei Zhang

**Affiliations:** 1 Key Laboratory of Tectonics and Petroleum Resources (China University of Geosciences), Ministry of Education, Wuhan 430074, China; E-Mails: hushzh@cug.edu.cn (S.H.); zdm2007@cug.edu.cn (D.Z.); 2 School of Earth Sciences and Engineering, Nanjing University, Nanjing 210093, China; E-Mail: jcao@nju.edu.cn (J.C.); wming0502@gmail.com (M.W.)

**Keywords:** diamondoid, oil maturity, biomarker, condensate, GC×GC-TOFMS, Junggar Basin

## Abstract

Diamondoids in crude oil are useful for assessing the maturity of oil in high maturation. However, they are very difficult to separate and accurately quantify by conventional geochemical methods due to their low abundance in oil. In this paper, we use comprehensive two-dimensional gas chromatography with time-of-flight mass spectrometry (GC×GC-TOFMS) to study the compounds in condensates from the Junggar Basin of northwest China and address their geological and geochemical applications. GC×GC-TOFMS improves the resolution and separation efficiency of the compounds. It not only separates the compounds that coelute in conventional GC-MS (e.g., 4, 8-dimethyl-diamantane and trimethyl-diamantane) but also allows the identification of compounds that were not previously detected (e.g., trimethyl-diamantane (15A)). A reversed-phase column system improves the separation capabilities over the normal phase column system. The diamondoid indexes indicate that a representative condensate from Well DX 10 is highly mature with equivalent Ro being approximately 1.5%.

## 1. Introduction

Diamondoids are rigid, three-dimensional cyclohexane-ring alkanes that have a diamond-like cage structure [1,2]. Diamondoids in crude oils and source rocks have extremely stable chemical properties. Once formed, they are strongly resistant to thermal degradation and destruction by microorganisms. Therefore, they are generally more stable than other hydrocarbon compounds in geological evolution [3,4]. Because of these factors, diamondoids have important geochemical implications in studying source rocks and oils (especially with high degree of thermal evolution), including maturity, biodegradation and organic facies. This area of research has been a highlight in petroleum geochemistry for many years [3,5–10].

However, the geological and geochemical implications of diamondoids are commonly difficult to address. This is because it is difficult to detect them simultaneously with conventional biomarkers in full scan during conventional GC-MS analysis. In addition, when using selective ion monitoring, they are still not easy to be identified due to their low boiling point and low concentration in oil (some are even in trace amounts). Furthermore, the measurement of diamondoids is conducted on saturated hydrocarbons. Unfortunately, the liquid chromatographic separation of saturated hydrocarbons may result in the loss of the diamondoids due to their low boiling point, which adds to the difficulty in the detection and quantitative analysis of the compounds.

In comparison with conventional GC-MS analysis, the method of comprehensive two-dimensional gas chromatography with time-of-flight mass spectrometry (GC×GC-TOFMS) is effective for separation and identification of complex mixtures, being characterized by high resolution, high sensitivity, large peak capacity, rapid analysis speed and regular qualitative analysis [11–17]. Using this method, diamondoids can be effectively distinguished simultaneously with conventional hydrocarbon compounds [18], providing good conditions for the study of diamondoids. In this paper, we used this method to analyze over 30 condensates collected from the western Junggar Basin of northwest China for the characterization of diamondoids and address their geological and geochemical implications.

## 2. Samples and Methods

The most representative condensate that has the best analytical results was collected from Well DX10 in the western Junggar Basin, with a depth at 3024–3048 m and a Carboniferous age. The condensate has a density of 0.7624 g/cm^3^, viscosity of 0.78 mPa.s, freezing point of −24.0 °C and wax content of 0.1%. The condensate was washed in a silica gel column by a solution consisting of dichloromethane and n-hexane in a proportion of 2:1. This aims to collect hydrocarbon fractions, which subsequently were condensed using a nitrogen evaporator and transferred for analysis. The silica gel is 100–200 mesh and was activated at 200 °C for 4 h.

We used a LECO GC×GC-TOFMS instrument system based on an Agilent 7890 N gas chromatograph fitted with a liquid nitrogen-cooled pulse jet modulator and a hot air pulse jet modulator. Each sample was injected in splitless mode. The injection volume was 0.2 μL and the temperature was 310 °C. The experimental system was verified with standard samples, which is prepared with *n*C_15_, *n*C_16_, *n*C_17_, *n*C_18_ and *n*C_19_ compounds.

Both normal phase column system and reversed phase column system were conducted. This aims at a comparative study. For the normal system, the inlet temperature was 310 °C, the first-dimension column was a nonpolar HP-5MS (60 m × 0.25 mm × 0.25 μm) that was held at 40 °C for 2 min and then ramped to 310 °C at 2 °C/min, and then maintained for 16 min. The modulator cold jet gas was dry N_2_, chilled with liquid N_2_. The modulator temperature offset was 30 °C higher than the main GC oven temperature. The modulation time is 6 s with a 1.5 s hot pulse time. Second dimension separations were performed using a DB-17HT (1.2 m × 0.1 mm × 0.1 μm) that was held at 45 °C for 2 min and then ramped to 315 °C at 2 °C/min, and then maintained for 16 min. The carrier gas was He at a constant flow rate of 1.5 mL/min.

For the reversed system, it consists of DB-17 (30 m × 0.25 mm × 0.25 μm) coupled with DB-5 (1.2 m × 0.1 mm × 0.1 μm). The inlet temperature was 300 °C, the first-dimension column was a polar DB-17 (30 m × 0.25 mm × 0.25 μm) that was held at 40 °C for 2 min and then ramped to 300 °C at 2 °C/min, and keep it for 7 min. The modulator cold jet gas was dry N_2_, being chilled with liquid N_2_. The modulator temperature offset was 30 °C higher than the main GC oven temperature. The modulation time is 7 s with a 1.7 s hot pulse time. Second dimension separations were performed using a DB-5HT (1.2 m × 0.1 mm × 0.1 μm) that was held at 45 °C for 2 min and then ramped to 305 °C at 2 °C/min, and then maintained for 7 min. The carrier gas was He at a constant flow rate of 1.0 mL/min.

The TOF-MS detector signal was sampled at 100 spectra/s and scan range of 55–550 amu. The transfer line held at a constant temperature of 280 °C. The TOF source temperature was 240 °C and the detector was set to −1475 V.

The software of Chromas TOF version 4.43 was used for data processing with database NIST 05. The data processing was mainly conducted by setting up several parameters in the software, such as baseline offset below (usually 0.5), peak width in first dimension (usually it is four times as the modulation time) and peak width in the second dimension (usually approximately 0.1 s) Under such conditions, the software combines fragment peaks whose similarity is over 700, which is an adjustable parameter and usually is 800 in the GC×GC study of oil geochemistry. By this, the software calculates the integrated peak areas automatically. Subsequently, the identification and recognition of compounds are realized by comparing the retention time of the NIST 05 database, combined with a comparison with a professional handbook of chromatograms and mass spectra of known petroleum and geological compounds [19–21].

## 3. Results and Discussion

Analytical results show that the diamondoid compounds identified in our work mainly include adamantanes and diamantanes. Their identification was obtained mainly according to comparison with the results of previous studies [2,7,22]. All the compounds elute in one run, demonstrating the advantage of the GC×GC study.

### 3.1. Identification of Adamantanes

**Figure 1:**
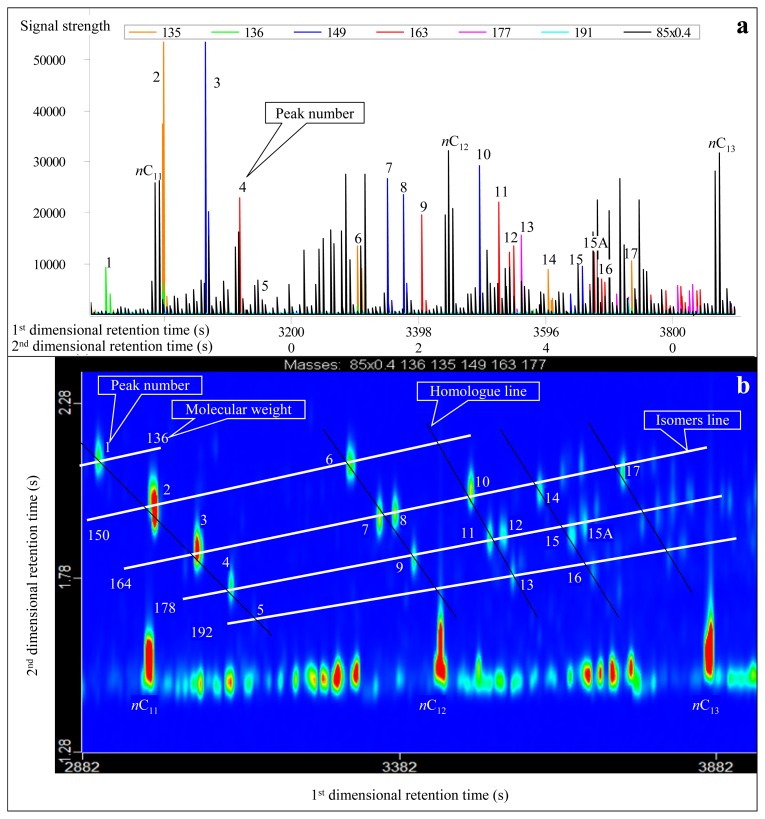
Real second-dimensional chromatogram (**a**) and GC×GC-TOFMS contour plot (**b**) of adamantanes identified in the condensate from Well DX 10 by GC×GC-TOFMS analysis under normal phase column system. The compounds are listed in Table 1.

The molecular formula of adamantanes is C*_n_*H_2_*_n_*_−4_ and the carbon number ranges from C_10_ to C_14_. Adamantanes were identified by characteristic fragment ions of *m/z* 136, 135, 149, 163, 177 and 191 (C_10_ to C_13_; Figure 1a). In the first-dimensional chromatogram, some adamantanes overlap with chain isoparaffins, such as *n*C_11_ and No.2 adamantane (Figure 1a). This leads to inaccurate quantitative analysis of adamantanes. In contrast, in GC×GC-TOFMS analysis, adamantanes can be effectively separated, although they have the same or similar first-dimensional retention time (Figure 1b). The carbon number of adamantanes that were identified in the condensate from Well DX 10 ranged from C_10_ to C_14_.

**Figure 2:**
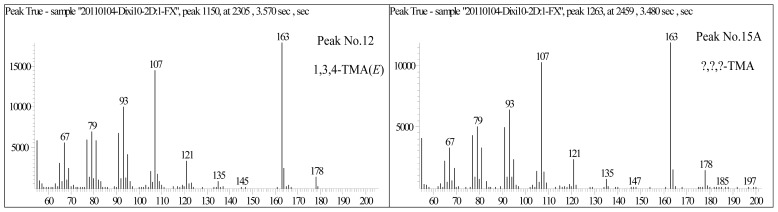
Mass spectra of 1,3,4-trimethyl-adamantane and No. 15A peak in Figure 1a.

In the trimethyl-adamantanes, there is a compound that has rarely been detected in conventional GC-MS analysis, which is located near the No. 15 peak (Figure 1a). It cannot be accurately named due to the lack of standard spectra and material so far. Since its chromatogram is very similar to the No. 15 peak (Figure 1a), we tentatively named it as the No. 15A peak. Its mass spectra is very similar to 1, 3, 4-trimethyl-adamantanes, with *m*/*z* 163 being the characteristic peak and molecular weight being 178 (Figure 2). Thus, it most likely belongs to the trimethyl-adamantane group of compounds. Furthermore, according to the peak order of multi-methyl-naphthalene and multi-methyl-phenanthrene, the peak of the compound in isomers shows a backward trend when the replacing position of multi-methyl becomes centralized. Therefore, we propose that the compound may be 1, 2, 3- or 2, 3, 4-trimethyladamantanes.

### 3.2. Identification of Diamantanes

**Figure 3:**
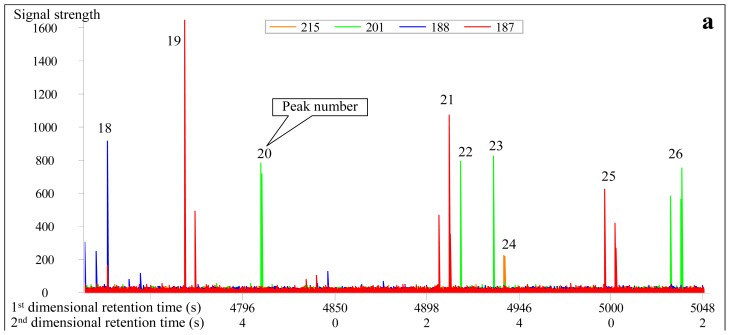

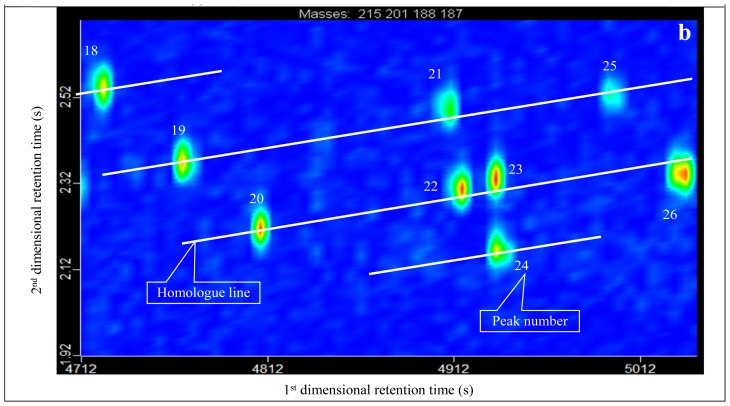
Real second-dimensional chromatogram (**a**) and GC×GC-TOFMS contour plot (**b**) of diamantanes identified in the condensate from Well DX 10 by GC×GC-TOFMS analysis under normal phase column system. The compounds are listed in Table 1.

Nine diamantane homologues were identified, ranging from C_15_ to C_17_ (No. 18 to 26 peaks in Figure 3a and Table 1), of which the No. 22 compound is the overlapped peak of 1,2-dimethyl-diamantane and 2,4-dimethyl diamantane. The No. 22, 23 and 24 peaks commonly coelute in conventional GC-MS analysis [18]. In contrast, in GC×GC-TOFMS analysis, the No. 23 and 24 peaks have the same first-dimensional retention time of 4934 s (Table 1). However, their second-dimension retention times are 2.164 s and 2.330 s, respectively, showing a difference of 0.166 s (Table 1). Thus, they can be preliminarily separated based on masses by first-dimensional analysis, and can be effectively separated by second-dimensional analysis (Table 1, Figure 3).

### 3.3. Difference between Normal and Reversed Phase Column Systems

**Figure 4:**
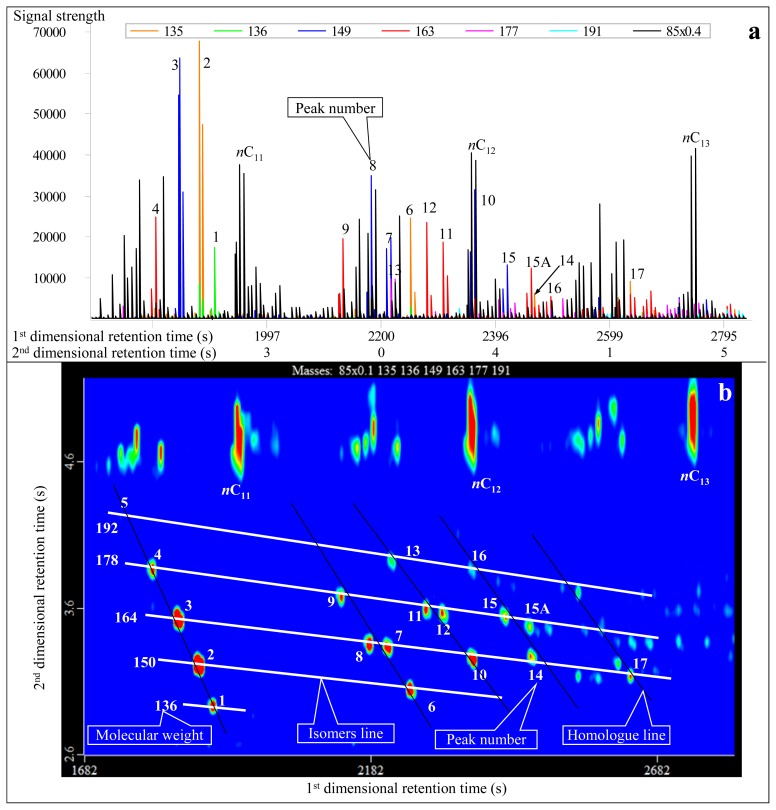
Real second-dimensional chromatogram (**a**) and GC×GC-TOFMS contour plot (**b**) of adamantanes identified in the condensate from Well DX 10 by GC×GC-TOFMS analysis under reversed phase column system. The compounds are listed in Table 1.

**Figure 5:**
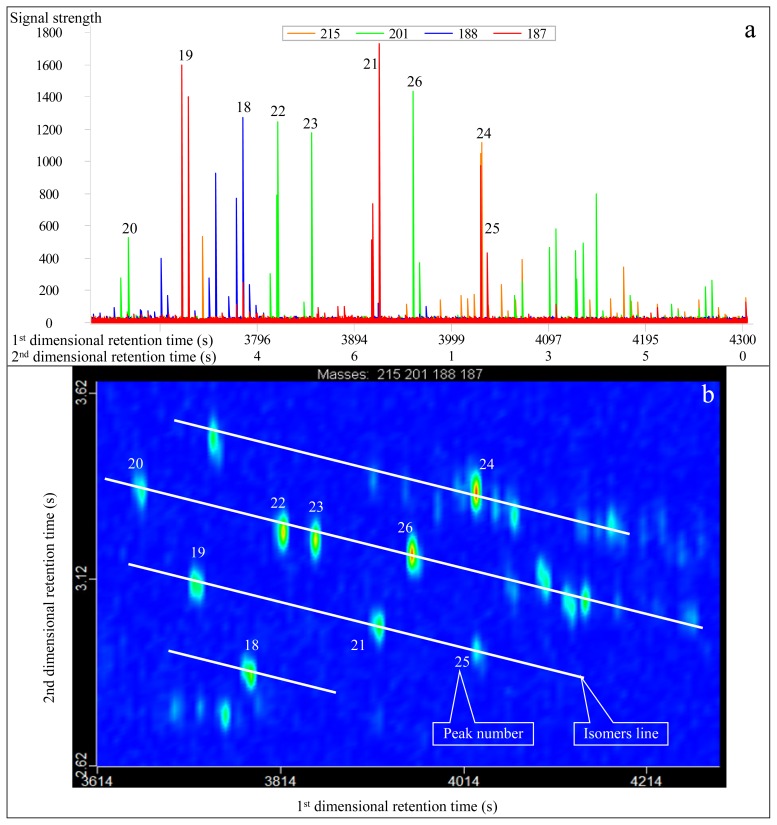
Real second-dimensional chromatogram (**a**) and GC×GC-TOFMS contour plot (**b**) of diamantanes identified in the condensate from Well DX 10 by GC×GC-TOFMS analysis under reversed phase column system. The compounds are listed in Table 1.

Analytical results conducted in normal and reversed phase column systems show differences (Table 1). In the normal system, the first-dimensional and second-dimensional retention times of the compounds with the same characteristic fragment ions increase simultaneously, showing a right-declined imbricate distribution (Figures 1 and 3). By contrast, in the reversed system, the second-dimensional retention time of the compounds decreases while the first-dimensional retention time increases, demonstrating a left-declined imbricate distribution (Figures 4 and 5). In the same second-dimensional chromatogram, the dispersion degree of compounds under the reversed system is obviously larger than that in the normal system, such as the No. 1 and 5 peaks in Table 1, whose distribution span is the largest. Although the second-dimensional retention time interval is only 0.47 s in the normal system, it is far greater in the reversed system (up to 1.28 s; Table 1, Figure 4). With respect to the second-dimensional retention time of the two pairs of cis trans isomers (No. 7 and No. 8, and No. 11 and No. 12), the difference in the reversed system is greater than that in the normal system (Table 1). As to the diamantanes, the first-dimensional retention time interval between No. 18 and 24 peaks is 210 s in the normal system, while it is 245 s in the reversed system (Table 1). In contrast, the second-dimensional retention time interval is 0.375 s and 0.480 s for the normal and reversed systems, respectively. Therefore, in summary, the identification of diamondoids is better in the reversed system (Figures 4 and 5) than in the normal system (Figures 1 and 3).

In our study, the normal and reversed column systems have similar experimental conditions in temperatures, pressure/flow of the carrier gas, *etc.* In contrast, the column length of the reversed system (30 m) is lower than that of the normal system (60 m). Nevertheless, the separation efficiency of the reversed system is still better than that of the normal system.

### 3.4. Geological and Geochemical Applications

As thermal stability of diamondoids varies with different positions of substituents, they can be used to constrain the degree of thermal evolution of source rocks and crude oils [2,6]. In particular, this is effective for oils with high maturation, which is commonly very hard to constrain in oil geological and geochemical studies [23]. In general, due to geochemical structures and natures, 1-methyl-adamantane (1-MA) is more stable than 2-methyl-adamantane (2-MA), and 4-methyl-diamantane (4-MD) is more stable than 1-methyl-diamantane (1-MD) and 3-methyl-diamantane (3-MD) [2]. Thus, the indexes I (1-MA/(1-MA + 2-MA)) andII (4-MD/(1-MD + 3-MD + 4-MD)) that are calculated based on peak area of the compounds increase with maturity, showing good correlations with *R*_o_ [2]. These two index values of the condensate from Well DX 10 based on analysis by normal and reversed phase column systems are 80.18% and 3%–45%, respectively, equivalent to an *R*_o_ of approximately 1.5%. This is difficult to determine by conventional geochemical analyses in previous studies. As stated previously, to quantitatively constrain the maturity of oil during its relatively high maturation stage is very difficult. Those commonly-used biomarker parameters that are effective in studying oil maturity under low to moderate maturation stage will have irregular and complex variations, e.g., C_29_ sterane 20S/(20S+20R), C_29_ sterane ββ/(ββ+αα) and Ts/(Ts+Tm) [23]. Thus, there are few studies on the quantitative characterization of the maturity of the DX 10 condensate.

## 4. Conclusions

1. Diamondoids can be effectively identified and characterized by GC×GC-TOFMS analysis, which is difficult to achieve using conventional GC-MS measurements. New trimethyl-adamantane 15A (the No. 15A peak in Table 1) compound was detected, which is present close to 1-methyl-3-ethyl-adamantane (the No. 15 peak in Table 1). It may be 1,2,3- or 2,3,4-trimethyladamantanes.
2. Diamondoids that cannot be effectively separated in conventional GC-MS measurements can be effectively separated in GC×GC-TOFMS analysis, such as 4, 8-dimethyl-diamantane (No. 23 peak in Table 1) and trimethyl-diamantane (No. 24 peak in Table 1). This leads to more accurate quantitative analysis and calculations.
3. The separation degree of diamondoids is affected by multiple factors and is generally better in a reversed phase column system than in a normal system.
4. According to diamondoid indexes indicative of maturity, the condensate from Well DX 10 is highly mature with equivalent R_o_ being approximately 1.5%, obtaining a new effective quantitative indicator. Thus, we obtained new understanding by GC×GC-TOFMS study on diamondoids, whose prospects in the study of petroleum geochemistry were shown.

## Figures and Tables

**Table 1:** Diamondoid compounds (adamantanes and diamantanes) identified in the condensate from Well DX 10 by GC×GC-TOFMS analysis.

Peak No.	Compound abbreviation	*m/z*	Molecular Weight	First-dimensional retention time (s)	Second-dimensional retention time (s)	Molecular Formula	Full name of compound
1	Adamantane (A)	136	136	2906 (1906*)	2.120 (2.940)	C_10_H_16_	Adamantane
2	1-MA	135	150	2996 (1878)	1.970 (3.230)	C_11_H_18_	1-Methyl-adamantane
3	1,3-DMA	149	164	3062 (1843)	1.860 (3.540)	C_12_H_20_	1,3-Dimethyl-adamantane
4	1,3,5-TMA	163	178	3116 (1801)	1.760 (3.870)	C_13_H_22_	1,3,5-Trimethyl-adamantane
5	1,3,5,7-TeTMA	177	192	3158 (1745)	1.650 (4.220)	C_14_H_24_	1,3,5,7-Tetramethyl-adamantane
6	2-MA	135	150	3302 (2249)	2.100 (3.050)	C_11_H_18_	2-Methyl-adamantane
7	1,4-DMA(*Z*)	149	164	3350 (2214)	1.950 (3.330)	C_12_H_20_	Cis-1,4-dimethyl-adamantane
8	1,4-DMA(*E*)	149	164	3374 (2179)	1.970 (3.360)	C_12_H_20_	Trans-1,4-dimethyl-adamantane
9	1,3,6-TMA	163	178	3404 (2130)	1.830 (3.680)	C_13_H_22_	1,3,6-Trimethyl-adamantane
10	1,2-DMA	149	164	3494 (2361)	2.030 (3.250)	C_12_H_20_	1,2-Dimethyl-adamantane
11	1,3,4-TMA(*Z*)	163	178	3524 (2305)	1.890 (3.570)	C_13_H_22_	Cis-1,3,4-Trimethyl-adamantane
12	1,3,4-TMA(*E*)	163	178	3548 (2277)	1.900 (3.600)	C_13_H_22_	Trans-1,3,4-Trimethyl-adamantane
13	1,2,5,7-TeTMA	177	192	3560 (2221)	1.790 (3.910)	C_14_H_24_	1,2,5,7-Tetramethyl-adamantane
14	1-EA	135	164	3602 (2459)	2.030 (3.280)	C_12_H_20_	1-Ethyl-adamantane
15	1-M-3-EA	149	178	3656 (2417)	1.900 (3.530)	C_13_H_22_	1-Methyl-3-ethyl-adamantane
15A	?,?,?-TMA	163	178	3674 (2459)	1.930 (3.480)	C_13_H_22_	?,?,?-Trimethyl-adamantane
16	1-E-3,5-DMA	163	192	3686 (2354)	1.800 (3.880)	C_13_H_22_	1-Methyl-3,5-dimethyl-adamantane
17	2-EA	135	164	3734 (2613)	2.080 (3.220)	C_12_H_20_	2-Ethyl-adamantane
18	Diamantane (D)	188	188	4724 (3782)	2.539 (2.870)	C_14_H_20_	Diamantane
19	4-MD	187	202	4766 (3719)	2.370 (3.110)	C_15_H_22_	4-Methyl-diamantane
20	4,9-DMD	201	216	4808 (3663)	2.220 (3.358)	C_16_H_24_	4,9-Dimethyl-diamantane
21	1-MD	187	202	4910 (3922)	2.491 (2.990)	C_15_H_22_	1-Methyl-diamantane
22	1,2-+2,4-DMD	201	216	4916 (3817)	2.309 (3.249)	C_16_H_24_	1,2-+2,4-Dimethyl-diamantane
23	4,8-DMD	201	216	4934 (3838)	2.330 (3.230)	C_16_H_24_	4,8-Dimethyl-diamantane
24	TMD	215	230	4934 (4027)	2.164 (3.350)	C**_17_**H_26_	Trimethyl-diamantane
25	3-MD	187	202	5000 (4027)	2.520 (2.935)	C_15_H_22_	3-methyl-diamantane
26	3,4-DMD	201	216	5036 (3957)	2.345 (3.190)	C_16_H_24_	2-Methyl-adamantane

* The corresponding retention time of reversed phase column system is listed in brackets.
